# The Aquaporin-3-Inhibiting Potential of Polyoxotungstates

**DOI:** 10.3390/ijms21072467

**Published:** 2020-04-02

**Authors:** Catarina Pimpão, Inês V. da Silva, Andreia F. Mósca, Jacinta O. Pinho, Maria Manuela Gaspar, Nadiia I. Gumerova, Annette Rompel, Manuel Aureliano, Graça Soveral

**Affiliations:** 1 Research Institute for Medicines (iMed.ULisboa), Faculty of Pharmacy, Universidade de Lisboa, 1649-003 Lisbon, Portugal; pimpaocatarina@gmail.com (C.P.); imvsilva@ff.ul.pt (I.V.d.S.); andreiafbm@medicina.ulisboa.pt (A.F.M.); jopinho@ff.ulisboa.pt (J.O.P.); mgaspar@ff.ulisboa.pt (M.M.G.); 2 Department of Biochemistry and Human Biology, Faculty of Pharmacy, Universidade de Lisboa, 1649-003 Lisbon, Portugal; 3 Department of Pharmaceutical Technology, Faculty of Pharmacy, Universidade de Lisboa, 1649-003 Lisbon, Portugal; 4 Universität Wien, Fakultät für Chemie, Institut für Biophysikalische Chemie, 1090 Vienna, Austria; nadiia.gumerova@univie.ac.at (N.I.G.); annette.rompel@univie.ac.at (A.R.); 5 Faculdade de Ciências e Tecnologia (FCT), CCMar, Universidade do Algarve, 8005-139 Faro, Portugal

**Keywords:** aquaporin, aquaglyceroporin, glycerol, polyoxotungstates, inhibitors, cancer, melanoma

## Abstract

Polyoxometalates (POMs) are of increasing interest due to their proven anticancer activities. Aquaporins (AQPs) were found to be overexpressed in tumors bringing particular attention to their inhibitors as anticancer drugs. Herein, we report for the first time the ability of polyoxotungstates (POTs), such as of Wells–Dawson P_2_W_18_, P_2_W_12_, and P_2_W_15_, and Preyssler P_5_W_30_ structures, to affect aquaporin-3 (AQP3) activity and impair melanoma cell migration. The tested POTs were revealed to inhibit AQP3 function with different effects, with P_2_W_18_, P_2_W_12_, and P_5_W_30_ being the most potent (50% inhibitory concentration (IC_50_) = 0.8, 2.8, and 3.2 µM), and P_2_W_15_ being the weakest (IC50 > 100 µM). The selectivity of P_2_W_18_ toward AQP3 was confirmed in yeast cells transformed with human aquaglyceroporins. The effect of P_2_W_12_ and P_2_W_18_ on melanoma cells that highly express AQP3 revealed an impairment of cell migration between 55% and 65% after 24 h, indicating that the anticancer properties of these compounds may in part be due to the blockage of AQP3-mediated permeability. Altogether, our data revealed that P_2_W_18_ strongly affects AQP3 activity and cancer cell growth, unveiling its potential as an anticancer drug against tumors where AQP3 is highly expressed.

## 1. Introduction

Polyoxometalates (POMs) are discrete metal oxo anions of transition metals such as Mo, W, V, and Nb amenable to a variety of structural transformations. In addition, other elements (most commonly P, Si, Al, As, Sb, etc.) may be included into these structures, or one of the addenda atoms may even be absent and/or substituted by other metals, such as Co, Ni, or Fe. Thus, POMs exhibit outstanding physical, chemical, and biochemical properties that remain to be completely understood and applied. Due to these properties, POMs are studied in environmental, chemical, and industrial fields, particularly in the areas of catalysis, corrosion prevention, smart glasses, and macromolecular crystallography, as well as in biology, such as for cancer treatment, bacterial infection, and diabetes, among others [1,2,3,4,5,6]. Although the number of POM studies with anticancer effects is increasing, their mechanisms of action in each type of cancer cell are still far from being understood [3]. The antitumor activity of these inorganic compounds seems to be caused, at least in part, by the inhibition of certain enzymes such as alkaline phosphatases, kinases, ecto-nucleotidases, glucosidases, and P-type ATPases [5,6,7,8]. Moreover, some POMs are also understood to interfere with mitochondria function and DNA, while others can exert an immunomodulatory activity [9,10]. As the incidence of cancer is increasing every year all over the world, along with the growing resistance effect and high toxicity of chemotherapeutic agents, some researchers chose POMs as alternative anti-tumor substances with promising results in suppressing tumor growth [3,9,11,12,13].

Several studies described the aberrant expression of aquaporins (AQPs) in different human cancer cells and tissues, frequently correlated with tumor stage and aggressiveness [14,15]. AQPs are highly conserved transmembrane proteins that facilitate the diffusion of water and glycerol across cell membranes, crucial for water and energy homeostasis [16]. The 13 human isoforms (AQP0–AQP12) currently described in humans are widely distributed among the body tissues and are classified according to their selectivity and sequence homology. Classical or orthodox aquaporins (AQP0, AQP1, AQP2, AQP4, AQP5, AQP6, AQP8) are mainly selective to water, aquaglyceroporins (AQP3, AQP7, AQP9, AQP10) facilitate glycerol and urea permeation in addition to water, and the selectivity of non-orthodox/S-aquaporins (AQP11 and AQP12) with lower homology, expressed in subcellular membranes, is still under study [17,18,19]. A few isoforms also facilitate the diffusion of H_2_O_2_ through cell membranes [20,21] and may impact cellular oxidative stress resistance and cancer progression [22,23]. The involvement of AQPs in cell proliferation and migration, tumor growth, and angiogenesis strongly supports their great potential as novel drug targets for cancer treatment [24,25]. In particular, the aquaglyceroporin AQP3 was found aberrantly expressed in various human cancers including human skin cell carcinomas and melanoma [26,27]. Malignant melanoma is one of the most invasive and metastatic human cancers frequently leading to fatal outcomes. Novel treatments that allow for the prevention and retardation of melanoma metastasis still need to be identified [26]. Studies reporting AQP3-modulating agents revealed metal compounds [28] and small molecules [29] as promising drug leads that deserve in vivo validation [25,30,31].

**Figure 1:**
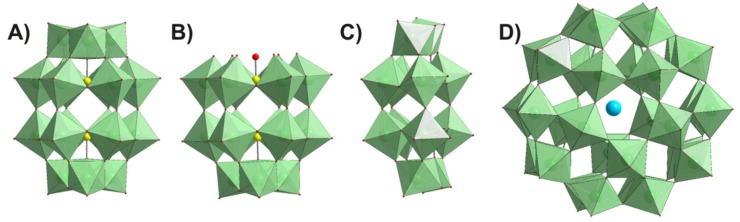
Polyhedral representation of polyoxotungstate (POT) structures tested in this study. (**A**) Wells–Dawson anion [P_2_W_18_O_62_]^6−^ (P_2_W_18_); (**B**) tri-lacunary Wells–Dawson anion [P_2_W_15_O_56_]^12−^ (P_2_W_15_); (**C**) hexa-lacunary Wells–Dawson anion [H_2_P_2_W_12_O_48_]^12−^ (P_2_W_12_); (**D**) Preyssler anion [NaP_5_W_30_O_110_]^14−^ (P_5_W_30_). Color code: {WO_6_}, mint; P, yellow; O, red; Na, blue.

This prompted us to investigate the possible effect of three Wells–Dawson and one Preyssler type polyoxotungstates (POTs) (Figure 1) on AQP3 activity and evaluate their potential as anticancer drugs for melanoma therapeutics. Herein, we screened, for the first time, POTs as inhibitors of AQP3-mediated membrane permeability in human red blood cells (hRBCs) that highly express AQP1 and AQP3, and further validated their selectivity in yeast cells transformed with human AQPs [32]. Furthermore, in human melanoma cells that showed high levels of AQP3 gene expression, we evaluated POT anticancer properties through their effect on cell viability and suppression of cell migration. Our data show that the solution of the P_2_W_18_ strongly affects AQP3 activity and cancer cell growth, unveiling its potential as an anticancer drug against tumors where AQP3 is highly expressed.

## 2. Results

### 2.1. Effect of Polyoxotungstates on the Permeability of Water and Glycerol

**Figure 2:**
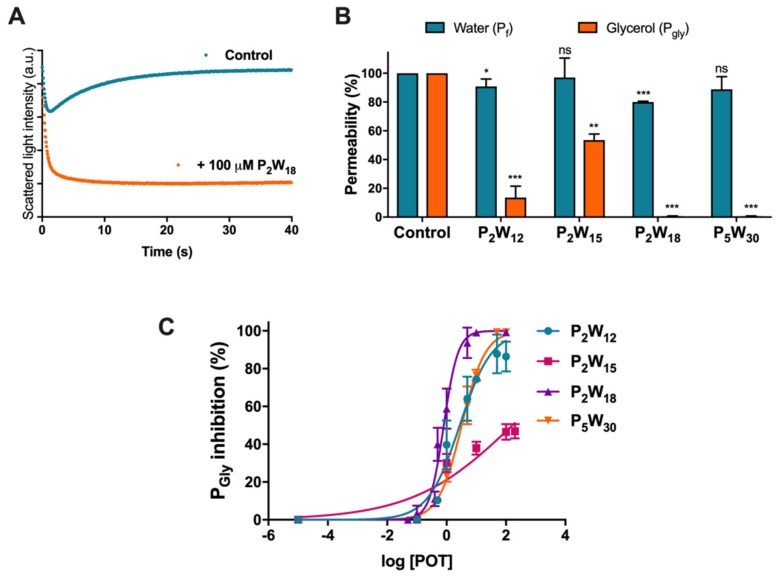
Effect of POTs on human red blood cell (hRBC) membrane permeability. (**A**) Representative stopped-flow signal showing changes in light scattering intensity when cells are confronted with a hyperosmotic glycerol solution. After a first shrinkage due to water efflux, cells reswell due to glycerol entrance via aquaporin 3 (AQP3) (control). Cell treatment with P_2_W_18_ prevents glycerol influx. (**B**) Water and glycerol permeability of hRBCs incubated with POTs (100 µM for 30 min). (**C**) Dose–response curves of glycerol permeability inhibition by POTs. Data are shown as means ± SD of three independent experiments. ns, non-significant; * *p* < 0.05; ** *p* < 0.01; *** *p* < 0.001. * treated vs. non-treated cells.

In order to assess the potential inhibitory activity of POTs on AQP3 function, we firstly studied the effects of four solutions of polyoxotungstates (POTs), K_6_[α-P_2_W_18_O_62_]·14H_2_O (abbreviated P_2_W_18_), Na_12_[α-P_2_W_15_O_56_]·24H_2_O (P_2_W_15_), K_12_[α-H_2_P_2_W_12_O_48_]·16H_2_O (P_2_W_12_), and (NH_4_)_14_[NaP_5_W_30_O_110_]·31H_2_O (P_5_W_30_)_,_ on water (P_f_) and glycerol permeability (P_gly_) in hRBCs, cells that largely express AQP1 (orthodox aquaporin) and AQP3 (aquaglyceroporin) [32], using stopped-flow spectroscopy. To evaluate water permeability, hRBCs incubated in isotonic phosphate-buffered saline (PBS) were challenged with a hyperosmotic sucrose solution (non-permeable solute), creating an osmotic gradient, which leads to fast water efflux and subsequent cell shrinkage. For glycerol permeability, cells were challenged with a hyperosmotic glycerol solution (permeable solute), inducing a fast cell shrinkage followed by cell reswelling due to water and glycerol influx via AQP3 [33] (Figure 2A). Water and glycerol permeability coefficients were calculated from the rate of cell volume change before and after treating cells with POTs (100 µM for 30 min) (Figure 2B).

As depicted, a strong inhibitory effect of glycerol permeability was observed for P_2_W_12_, P_2_W_18_, and P_5_W_30_, while P_2_W_15_ revealed a low potency in inhibiting the AQP3-mediated glycerol transport. In addition to glycerol, both P_2_W_18_ and P_2_W_12_ also affected cell water permeability (P_f_) but to a minor extent. Since AQP3 has both water and glycerol channeling activity, this small decrease in P_f_ indicates a full blockage of the AQP3 channel (Figure 2B). Subsequently, we performed permeability assays with POTs concentrations ranging from 0.1 to 100 µM. The dose–response curves of the tested POTs demonstrate their AQP3 inhibitory potency (Figure 2C and Table 1), showing P_2_W_15_ with the largest 50% inhibitory concentration (IC_50_) value and lowest effect (*p* < 0.001). Although both P_2_W_18_ and P_5_W_30_ displayed the highest values of P_gly_ inhibition (99.24% ± 0.03% and 99.31% ± 0.14%, respectively), P_2_W_18_ exhibited the lowest IC_50_ value (0.80 ± 0.04 µM) from all the tested POTs (*p* < 0.001) (Table 1), revealing it to be the most potent AQP3 inhibitor in this series. P_2_W_12_ also showed a low IC_50_ value (2.78 ± 0.09 µM), but higher than P_2_W_18_ and non-significantly different from P_5_W_30_.

**Figure 3:**
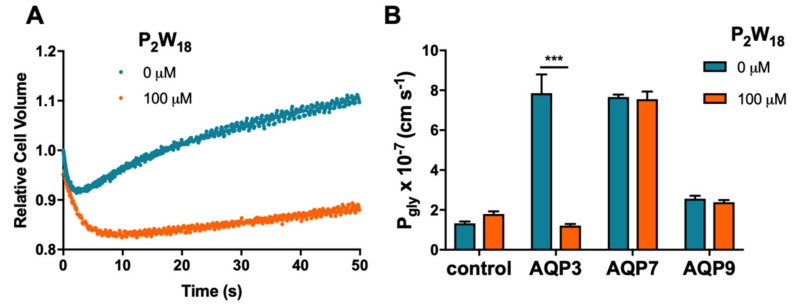
Effect of P_2_W_18_ on human aquaglyceroporins expressed in yeast. (**A**) Change in relative cell volume of AQP3-expressing cells challenged with a glycerol osmotic gradient. (**B**) Glycerol permeability (P_gly_) of yeast cells transformed with the empty vector (control) or expressing human AQP3, AQP7, or AQP9, non-treated and treated with 100 µM P_2_W_18_ for 30 min. Data are shown as means ± SD of three independent experiments. *** *p* < 0.001, treated vs. non-treated cells.

To validate the results above that showed P_2_W_18_ as a potent AQP3 inhibitor and to evaluate its selectivity, this compound was tested in the yeast *Saccharomyces cerevisiae* model, previously optimized by us and used for heterologous aquaporin functional studies [23,34,35,36]. Yeast cells, depleted of endogenous aquaporins, were transformed with either the empty plasmid (control cells) or the plasmid encoding human aquaglyceroporins (AQP3, AQP7, and AQP9). For permeability assays, cells were loaded with the volume-sensitive dye CFDA and were challenged with a hyperosmotic glycerol solution to evaluate glycerol permeability [34] (Figure 3A). For inhibition assays, cells were previously incubated with P_2_W_18_ (100 µM, 30 min). Figure 3B shows that P_2_W_18_ strongly inhibited AQP3-mediated glycerol transport, whereas P_gly_ of cells expressing AQP7 or AQP9 was not affected when compared with non-treated cells. Given the lack of effect on AQP7- and AQP9-mediated glycerol permeability, P_2_W_18_ can be considered selective for the aquaglyceroporin AQP3 isoform.

### 2.2. Effect of Polyoxotungstates on Melanoma Cell Migration

**Figure 4:**
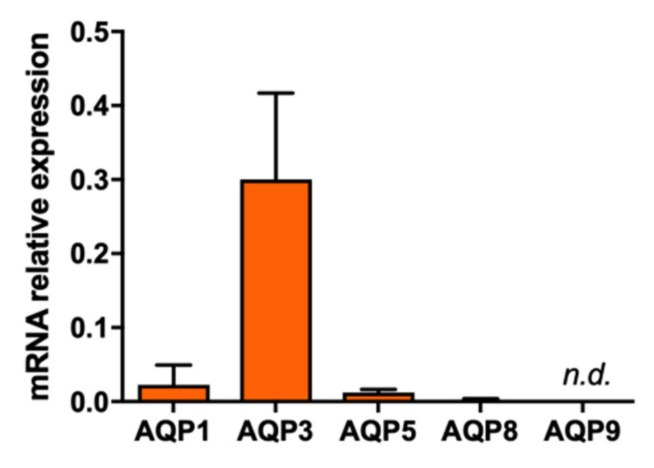
Screening AQPs expression in human melanoma cells. AQP messenger RNA (mRNA) expression in MNT-1 cells normalized to the mean of two housekeeping genes, *HTRP-1* and *β-actin*, showing AQP3 as the most expressed isoform. Data represent means ± SD of three independent experiments. n.d., not detected.

To investigate the relevance of inhibiting AQPs in melanoma cancer progression, the expression of AQP isoforms involved in cancer was firstly screened in MNT-1 cells by quantitative PCR. As depicted in Figure 4, AQP3 is the most expressed isoform in human melanoma MNT-1 cells, as reported for human skin tumors [27]. AQP1, AQP5, and AQP8 are also expressed in these cells although at lower levels, while AQP9 was not detected.

**Figure 5:**
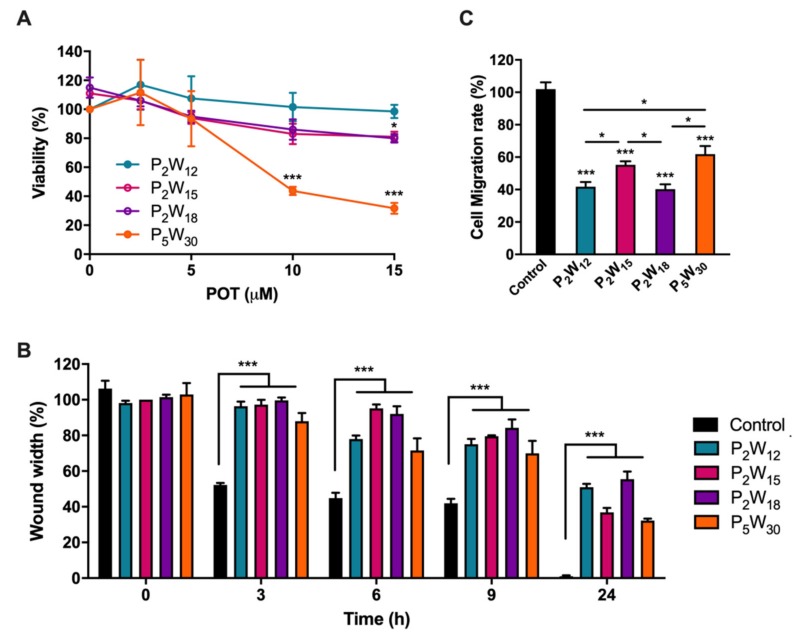
Effect of POTs on viability and migration of human melanoma cells. (**A**) Cell viability determined by 3-(4,5-dimethylthiazolyl-2)-2,5-diphenyltetrazium bromide (MTT) assay after cell exposure to a range of the four POT concentrations for 24 h. (**B**) Wound closure progression in cells non-treated (control) and treated with 5 µM POTs at 0, 3, 6, 9, and 24 h. (**C**) Cell migration of cells non-treated and treated with 5 µM POTs. Results are expressed as means ± SD of three independent experiments. * *p* < 0.05, *** *p* < 0.001, treated vs. non-treated cells and between POTs.

To further investigate whether AQP3 inhibition by POTs underlies signal transduction mechanisms that affect melanoma progression, we evaluated the rate of melanoma cell migration before and after treatment with POTs. Previous to the migration assay, we evaluated the POT effect on MNT-1 cell viability to define the most suitable concentration to be tested in migration without compromising cell viability (Figure 5A). The four POTs were tested at different concentrations, and cell viability was measured after 24 h of incubation with the compounds. 

As shown, treatment with up to 15 μM P_2_W_12_ proved to be harmless, while, for P_2_W_15_ and P_2_W_18_, around 20% loss of cell viability was observed. For P_5_W_30_, the higher concentrations were shown to be cytotoxic (Figure 5A). Thus, in subsequent cell migration assays, POTs were tested at 5 µM, a concentration above the IC_50_ value that assures AQP3 inhibition and >90% cell viability. 

Cell migration was carried out by a wound closure assay followed at 0, 3, 6, 9, and 24 h (Figure 5B). All compounds delayed melanoma cell migration compared to the control condition where the wound was totally closed in less than 24 h (Figure 5B and Supplementary Materials Figure S5). P_2_W_18_ exhibited the strongest inhibitory effect on cell migration (64%), followed by P_2_W_12_ (55%)_,_ P_2_W_15_ (50%), and P_5_W_30_ (39%) (Figure 5C).

## 3. Discussion

This study describes, for the first time, that POTs can affect aquaporin function and lead to the inhibition of cancer cell migration. POMs are potential candidates for anticancer drugs in the future since several types of POMs showed anticancer activities at various concentrations ranging from nM to µM [3,9,13,37,38,39,40,41,42,43,44]. In fact, in most cases, when compared with already approved anticancer drugs, POMs were more efficient. Indeed, one of the first studies showed that the polyoxomolybdate [NH_3_Pr]_6_[Mo_7_O_24_] presented antitumor activity against various human cancer cell lines and human cancer xenografts, with higher inhibitory rates than already approved drugs, such as cisplatin and fluorouracil [42,43,44]. 

AQPs and, in particular, the AQP3 isoform are well known to be involved in cancer biology. AQP3 is related to wound healing, lipid metabolism, and regulation of the proliferation and differentiation of several cancer cell types. The different mechanisms proposed to explain AQP3 participation in tumor growth and spread include its ability to transport glycerol, a key molecule for metabolic reactions in energy demanding cancer cells [14], as well as the ability to transport H_2_O_2_, modulating oxidative stress and triggering signaling cascades responsible for cell proliferation and migration [23,45]. In this regard, AQP-targeted drugs are believed to have great potential in treating cancer, where metallic compounds are considered the most potent AQP3 inhibitors. To date, to the best of our knowledge, no POMs were tested on AQPs activity. Therefore, this pioneering study reveals that some POTs, such as presented in solution of Wells–Dawson P_2_W_18_ and Preyssler P_5_W_30_ phosphotungstates, are potent inhibitors of AQP3, reducing the glycerol permeability to almost 0%. For the solutions of lacunary Wells–Dawson POTs, P_2_W_12_ and P_2_W_15_, lower inhibitory potency was observed (about 86% and 46%), with minimal or no effect on water fluxes, while the solution of intact P_2_W_18_ additionally induced 20% of water inhibition (Figure 2), which may represent a full blockage of the AQP3 channel. In fact, knowing that, in hRBCs, only 10% of water fluxes occur by passive diffusion via the lipid bilayer, with the remaining 90% channeled by aquaporins [46], and knowing that AQP1 is the one responsible for the main bulk of water flow, the smaller 20% decrease in water permeability suggests that AQP3 is the only isoform attained by P_2_W_18_. Interestingly, the same degree of inhibition was detected for the gold compound Auphen and, in general, all the gold series, known as selective AQP3 inhibitors, which exhibited a modest effect on water permeability (~15% to 20%) while drastically decreasing glycerol transport in the same cell system [28,47]. The fact that P_2_W_18_ exhibits an IC_50_ value in the nanomolar range (0.8 ± 0.04 μM) reinforces its potent inhibitory effect against AQP3, being comparable to the gold complexes already recognized as potent inhibitors of AQP3 [28,48]. These results were further validated by testing P_2_W_18_ in yeast cells individually expressing three human aquaglyceroporins (AQP3, AQP7, and AQP9), where the solution of P_2_W_18_ was confirmed to block near 100% glycerol permeability and showed selectivity for AQP3, displaying a lack of inhibitory effect on AQP7- and AQP9-expressing cells. Considering that gold compounds affect both AQP3 and AQP7 [36,49], this POT may be advantageous as a drug candidate for AQP3-overexpressing cancers. Thus, in addition to the effects on AQP3 activity, we further analyzed the effects of POTs in melanoma cell migration. 

The few studies addressing the effect of POMs in melanoma, with most of them using B16 murine melanoma cells, reported a decrease in cell viability and antitumor activity [50,51], as well as impaired cellular tyrosinase activity and melanin formation [52]. More recently, encapsulated polyoxomolybdates and POTs were also studied in murine melanoma [52,53]. Selective cytotoxic tendencies were observed against murine cells causing necrotic cell death, probably due to the disruption of plasma membranes and antiproliferative cell cycle arrest in the gap (G_0_/G_1_) and G_2_/mitosis (M) phases [53]. In the present study, we firstly confirmed the highest expression level of AQP3 in a human melanoma cell line, in agreement with the human tumor phenotype. Subsequently, we investigated if the POTs under study, in addition to the AQP3-inhibitory effect, would affect melanoma cell migration. Interestingly, all the tested POTs caused a reduction of the migration rate; however, again, the solution of P_2_W_18_ presented the strongest effect. It is worth mentioning that, in a physiological pH range as required for optimal activity of AQP3 [35] and for most other biological investigations, the Wells–Dawson and Preyssler POTs should not be considered as intact species and have to be expected to undergo at least partial hydrolysis. The careful speciation of respective POT clusters at physiological pH 7.4 (PBS medium) and in water by ^31^P-NMR analysis to address cluster species responsible for interaction in solution was performed (Supplementary Materials Figures S1–S3). Solutions of the most promising AQP3 inhibitor, P_2_W_18_, contain intact Wells–Dawson anions, along with the mono-lacunary [P_2_W_17_O_61_]^10−^ anion (Supplementary Materials Figures S1, S3, and S4). The analysis of NMR spectra of P_5_W_30_ solution revealed that, immediately after dissolving the Preyssler POT, three anions are present in solution: intact Preyssler, intact Dawson, and mono-lacunary Dawson anions (Supplementary Materials Figure S2). However, during the incubation of P_5_W_30_ solution at 37 °C, the content of P_2_W_18_ decreased and, after 24 hours, only intact Preyssler and mono-lacunary Dawson anion were present in solution (Supplementary Materials Figures S1 and S2). The spectra of P_2_W_12_ and P_2_W_15_ have signals corresponding to POT anions of lower intensities due to their poor solubility. Interestingly, in the solution of P_2_W_12_, the intact Preyssler anion was detected (Supplementary Materials Figure S1). At the same time, after dissolving tri-lacunary Wells–Dawson POT, only mono-lacunary species are present in PBS buffer (Supplementary Materials Figure S1). Although more investigations are needed to establish structure–activity relationships, it can nevertheless be assumed that it is the intact phosphotungstate that is responsible for the observed inhibitory effect on AQP3 activity and melanoma cell migration.

## 4. Materials and Methods

### 4.1. Polyoxometalates

The polyoxometalates used in this study, K_6_[α-P_2_W_18_O_62_]·14H_2_O (abbreviated P_2_W_18_), K_12_[α-H_2_P_2_W_12_O_48_]·16H_2_O (P_2_W_12_), Na_12_[α-P_2_W_15_O_56_]·24H_2_O (P_2_W_15_), and (NH_4_)_14_[NaP_5_W_30_O_110_]·31H_2_O (P_5_W_30_) (Figure 1, Table 2), were synthesized according to published procedures [54,55], and their purity was confirmed by infrared spectroscopy. Stock solutions of POTs were freshly prepared by dissolving the solid compound in water and keeping the solution at low temperature to avoid POT decomposition. The concentrations of the stock solutions were 10 mM and/or 1 mM for all POTs. 

For solution stability studies, ^31^P-NMR spectroscopy was used. ^31^P-NMR spectra were recorded with a Bruker FT-NMR spectrometer Avance Neo 500 MHz (Bruker, Rheinstetten, Germany) at 25 °C and 202.53 MHz. Chemical shifts were measured relative to 85% H_3_PO_4_.

### 4.2. Ethics Statement

Venous blood samples were obtained from healthy human volunteers following a protocol approved by the Ethics Committee of the Faculty of Pharmacy of the University of Lisbon (Instituto Português de Sangue Protocol SN-22/05/2007). Informed written consent was obtained from all participants.

### 4.3. Erythrocyte Sampling and Preparation

Venous blood samples were collected from anonymous human donors in citrate anticoagulant (2.7% citric acid, 4.5% trisodium citrate, and 2% glucose) to prevent coagulation. The blood was centrifuged at 750× *g* for 10 min at room temperature (RT) to isolate the erythrocytes. After washing three times with PBS (NaCl 137 mM, KCl 2.7 mM, Na_2_HPO_4_ 10 mM, KH_2_PO_4_ 1.8 mM, pH 7.4), hRBCs were diluted to a 0.5% suspension and kept on ice to be immediately used in the experiments. 

### 4.4. Strains, Plasmids, and Growth Conditions

Human aquaporin (AQP3, AQP7, and AQP9) complementary DNA (cDNA) was PCR-amplified from the pWPi-DEST plasmid [49] and C-terminally fused to a green fluorescent protein (GFP) of the centromeric plasmid, pUG35 [60]. *Escherichia coli* DH5α was used as a host for routine propagation, and plasmids were purified with a GenElute^TM^ Plasmid Miniprep Kit (Sigma-Aldrich, St. Louis, MO, USA). *E. coli* transformants were maintained and grown in Luria–Bertani broth (LB) with ampicillin (100 µg∙mL^−1^) at 37 °C [36]. For functional studies, *Saccharomyces cerevisiae*, 10560-6B MATα leu2::hisG trp1::hisG his3::hisG ura352 aqy1D::KanMX aqy2D::KanMX (YSH1770) was used as a host strain for heterologous expression of AQP3, AQP7, and AQP9. Yeast cultures were grown at 28 °C with orbital shaking in YNB (yeast nitrogen base, DIFCO) without amino acids, 2% (*w*/*v*) glucose supplemented with the adequate requirements for prototrophic growth [61]. For subcellular localization of GFP-tagged AQP3, AQP7, and AQP9 in *S. cerevisiae*, yeast transformants in the mid-exponential phase were observed with a Zeiss Axiovert 200 fluorescence microscope, at 495 nm excitation and 535 nm emission wavelengths, confirming more than 80% localization at the yeast plasma membrane.

### 4.5. Permeability Assays

Light scattering and fluorescence stopped-flow spectroscopy was used to monitor cell volume changes of RBC [28] and yeast transformants loaded with the concentration-dependent self-quenching fluorophore 5-(and-6)-carboxyfluorescein diacetate (CFDA, 1 mM, 10 min at 30 °C) [34], respectively. Experiments were performed on a HI-TECH Scientific PQ/SF-53 stopped-flow apparatus, which has a 2-ms dead time and is temperature-controlled, interfaced with an IBM PC/AT compatible 80386 microcomputer. After challenging cell suspensions with an equal volume of shock solution at 23 °C, the time course of volume change was measured by following the 90° scattered light intensity at 400 nm or fluorescence intensity (excitation 470 nm and emission 530 nm). For each experimental condition, 5–7 replicates were analyzed. Baselines were acquired using the respective incubation buffers as isotonic shock solutions. 

For osmotic water permeability (P_f_) measurements, a hyperosmotic shock solution containing a non-permeable solute was used (for RBC assays, sucrose 200 mM in PBS pH 7.4; for yeast assays, sorbitol 2.1 M in K^+^ citrate pH 7.4) producing an inwardly directed gradient of the solute. For glycerol permeability (P_gly_) measurements, a hyperosmotic shock solution containing glycerol was used (for RBC assays, glycerol 200 mM in PBS pH 7.4; for yeast assays, glycerol 2.1 M in K^+^ citrate pH 7.4) creating an inwardly directed glycerol gradient. After the first fast cell shrinkage due to water outflow, glycerol influx in response to its chemical gradient was followed by water influx with subsequent cell re-swelling. 

For hRBCs, P_f_ was estimated by P_f_ = k (V_o_/A)(1/V_w_(osm_out_)_∞_), where V_w_ is the molar volume of water, V_o_/A is the initial cell volume to area ratio, (osm_out_)_∞_ is the final medium osmolarity after the applied osmotic gradient, and k is the single exponential time constant fitted to the light scattering or fluorescence signal of yeast [34] or RBC shrinkage [28]. P_gly_ was calculated by P_gly_ = k (V_o_/A), where V_o_/A is the initial cell volume to area ratio, and k is the single exponential time constant fitted to the light scattering signal of glycerol influx in erythrocytes. 

For yeast cells, fluorescent glycerol traces obtained were corrected by subtracting the baseline slope that reflects the bleaching of the fluorophore. Optimization of yeast permeability values was accomplished by numerical integrations using a mathematical model implemented in the Berkeley Madonna software (http://www.berkeleymadonna.com/) as described [36].

To assess the effect of the polyoxotungstates, cells were incubated with different concentrations of these compounds for 30 min at RT before stopped-flow experiments. The inhibitor concentration that corresponds to 50% inhibition (IC_50_) was calculated by nonlinear regression of dose–response curves (GraphPad Prism software) using the following equation: *y* = 100/(1 + 10^((LogIC50 − *x*)^ × HillSlope)), where HillSlope describes the steepness of the family of curves.

### 4.6. Cell Culture

Human melanoma cells (MNT-1; RRID: CVCL_5624) were cultured in Dulbecco’s modified Eagle’s medium (DMEM) with high glucose (4.5 g/L), supplemented with 10% fetal bovine serum and 100 IU/mL of penicillin and 100 µg/mL streptomycin (complete medium) at 37 °C under a 5% CO_2_ atmosphere.

### 4.7. RNA Isolation, cDNA Synthesis, and Quantitative PCR

Total RNA was extracted from cultured cells using TRIzol Reagent (Invitrogen), according to the manufacturer’s protocol. RNA was quantified and qualified with Nanodrop 2000c spectrophotometer to ensure optimal quality (260/280 and 260/230 around 2). For template cDNA synthesis, 1 µg of total RNA was reverse-transcribed in a 20-µL final volume using the first-strand cDNA synthesis kit (NZYtech, Lisbon, Portugal) according to the manufacturer’s protocol. Real-time PCR reactions were carried out using a CFX96 Real-Time System C1000 (BioRad, Hercules, CA, USA), the TaqMan Universal PCR Master Mix (Applied Biosystems Thermo Fisher Scientific, Waltham, MA, USA), and the following specific TaqMan pre-designed gene expression primers: AQP1 (Hs01028916_m1), AQP3 (Hs01105469_g1), AQP5 (Hs00387048_m1), AQP8 (Hs01086280_g1), AQP9 (Hs00175573_m1), HPRT-1 (Hs02800695_m1), and ACTB (Hs99999903_m1) (Applied Biosystems, ThermoFisher Scientific, Waltham, MA, USA). The cDNA was amplified in the following conditions: 50 °C for 2 min, 95 °C for 10 min, followed by 45 cycles of 15 s at 95 °C and 1 min at 60 °C.

The relative quantification of gene expression was determined using the 2^Ct^ method based on a Livak method modification [62]. AQP gene expression was normalized to the mean of two housekeeping genes (HPRT-1 and β-actin) [63]. This procedure was performed in triplicate.

### 4.8. Viability Assay

Cell viability was evaluated by 3-(4,5-dimethylthiazolyl-2)-2,5-diphenyltetrazium bromide (MTT) assay. For that purpose, cells were inoculated at a density of 50,000 cells∙cm^−2^ in 96-well plates and grown at 37 °C, 5% CO_2_ until reaching 80% confluence. Cells were then incubated for 24 h with P_2_W_12_, P_2_W_15_, P_2_W_18_, and P_5_W_30_ at different concentrations (0, 2.5, 5, 10, and 15 µM). After incubation, cells were washed with PBS and incubated with MTT reagent 0.5 mg∙mL^−1^ in PBS for 3 h at 37 °C, 5% CO_2_. Finally, purple formazan crystals were solubilized with DMSO for 10 min, and the absorbance was measured at 570 nm. This procedure was performed in triplicate.

### 4.9. Migration Assay

MNT-1 cells were inoculated in 12-well plates at a density of 50,000 cells∙cm^−2^ and were allowed to adhere and grow until 80–90% confluence. The cell monolayer was wounded with an even trace using a sterile 10-µL tip and washed with PBS to remove debris. Cells were incubated with 5 µM P_2_W_12_, P_2_W_15_, P_2_W_18_, or P_5_W_30_ (diluted in DMEM medium) at 37 °C, 5% CO_2_. Images of the wound closure were captured every 3 h under a light microscope and analyzed using the software ImageJ (https://imagej.net). Wound closure was normalized to initial wound area at time 0. This procedure was performed in triplicate.

### 4.10. Statistical Analysis

All the experiments were performed in three biological and at least two technical triplicates. Data are presented as means ± standard deviation (SD). Statistical analysis between groups was performed by two-way ANOVA and non-parametric Mann–Whitney test. A *p*-value <0.05 was considered statistically significant. Statistical analyses were performed using the Graph Prism software (GraphPad Software).

## 5. Conclusions

Summing up, we demonstrate that POTs affect the activity of AQP3 and impair the migration rate of melanoma cells, with P_2_W_18_ being the most potent. P_2_W_12_ and P_5_W_30_ demonstrate similar, but higher IC_50_ values, while remaining promising inhibitors. The anticancer properties of these compounds may be in part due to the blockage of AQP3-mediated permeability, unveiling their potential as anticancer drugs against melanoma tumors.

## Figures and Tables

**Table 1:** Maximal inhibition and 50% inhibitory concentration (IC_50_) values of AQP3 glycerol permeability inhibition by POTs.

POTs	Glycerol Permeability	
	Max Inhibition (%)	IC50 (µM)
P_2_W_12_	86.40 ± 7.91	2.78 ± 0.09
P_2_W_15_	46.47 ± 4.18	>100
P_2_W_18_	99.24 ± 0.03	0.80 ± 0.04
P_5_W_30_	99.31 ± 0.14	3.24 ± 0.03

**Table 2:** POTs used in this study.

Formula	Abbreviation	Net Charge	Charge Density (Charge/Number of Addenda Atoms Ratio)	Synthesized According to	First Structural Report in	^31^P-NMR Signals Assignment According to
K_6_[α-P_2_W_18_O_62_]·14H_2_O	P_2_W_18_	−6	0.33	[54]	[56]	[54]
K_12_[α-H_2_P_2_W_12_O_48_]·16H_2_O	P_2_W_12_	−12	1	[54]	[57]	[54]
Na_12_[α-P_2_W_15_O_56_]·24H_2_O	P_2_W_15_	−12	0.8	[54]	[58]	[54]
(NH_4_)_14_[NaP_5_W_30_O_110_] 31H_2_O	P_5_W_30_	−14	0.47	[55]	[59]	[55]

## Supplementary Materials

Supplementary materials can be found at https://www.mdpi.com/1422-0067/21/7/2467/s1.

## Abbreviations

AQP	Aquaporin
hRBC	Human red blood cells
P_f_	Water permeability coefficient
P_gly_	Glycerol permeability coefficient
POMs	Polyoxometalates
POTs	Polyoxotungstates
