# Supplementary material for: The Aquaporin-3-Inhibiting Potential of Polyoxotungstates
Source: Int J Mol Sci. 2020 Apr 2;21(7):2467. doi: 10.3390/ijms21072467 (PMC7177757; doi:10.3390/ijms21072467)
Supplement: Supplementary file 1 [file ijms-21-02467-s001.pdf]

SUPPLEMENTARY INFORMATION FOR

The aquaporin-3 inhibiting potential of polyoxotungstates

Catarina Pimpão [1,2,†], Inês V. da Silva [1,2,†], Andreia F. Mósca [1,2], Jacinta O. Pinho [1,3], Maria Manuela Gaspar [1,3], Nadiia I. Gumerova [4], Annette Rompel [4], Manuel Aureliano [5,*] and Graça Soveral [1,2,*]

[1] Research Institute for Medicines (iMed.ULisboa), Faculty of Pharmacy, Universidade de Lisboa, 1649-003 Lisboa, Portugal; pimpaocatarina@gmail.com; imvsilva@ff.ul.pt; andreiafbm@medicina.ulisboa.pt; jopinho@ff.ulisboa.pt; mgaspar@ff.ulisboa.pt; gsoveral@ff.ulisboa.pt
[2] Department of Biochemistry and Human Biology, Faculty of Pharmacy, Universidade de Lisboa, 1649-003 Lisboa, Portugal
[3] Department of Pharmaceutical Technology, Faculty of Pharmacy, Universidade de Lisboa, 1649-003 Lisboa, Portugal
[4] Universität Wien, Fakultät für Chemie, Institut für Biophysikalische Chemie, 1090 Vienna, Austria; nadiia.gumerova@univie.ac.at; annette.rompel@univie.ac.at
[5] Faculdade de Ciências e Tecnologia (FCT), CCMar, Universidade do Algarve, 8005-139 Faro, Portugal; maalves@ualg.pt
† authors contributed equally to this work
* Correspondence: gsoveral@ff.ulisboa.pt, Tel: +351-217-946-461 (G.S.); maalves@ualg.pt, Tel: +351-289-900-805 (M.A.)

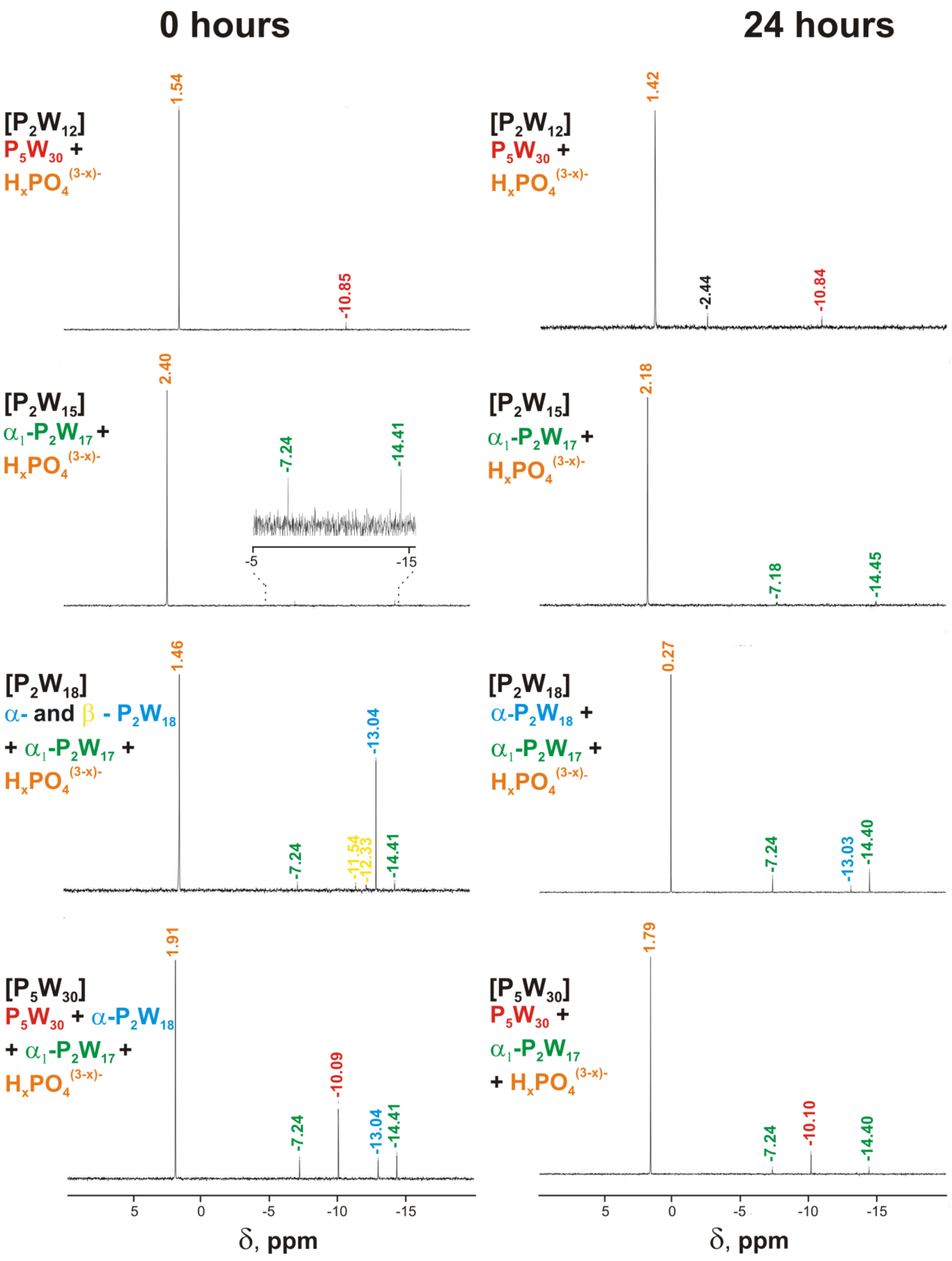

Figure S1. ^{31}P-NMR spectra of $\mathbf{P_5W_{30}}$, $\mathbf{P_2W_{18}}$, $\mathbf{P_2W_{12}}$ and $\mathbf{P_2W_{15}}$ in PBS medium at pH 7.4, recorded after preparation (0 hours) and after 24 hours of incubation at 37 °C. In phosphate formula x = 0-2.

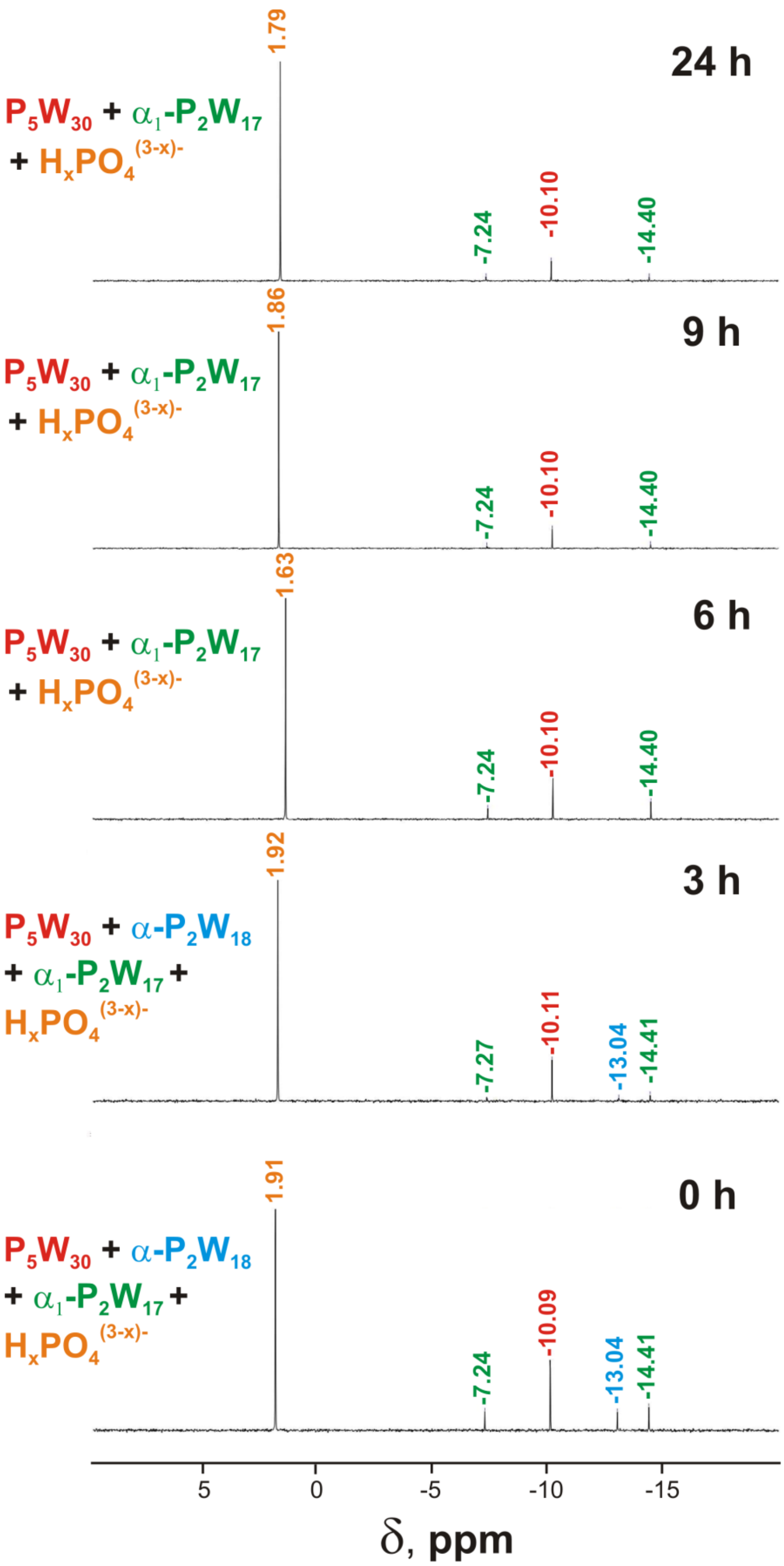

Figure S2. ^{31}P-NMR spectra of **P_5W_{30}** in PBS medium at pH 7.4, recorded after different time intervals of incubation at 37 °C. In phosphate formula x = 0-2.

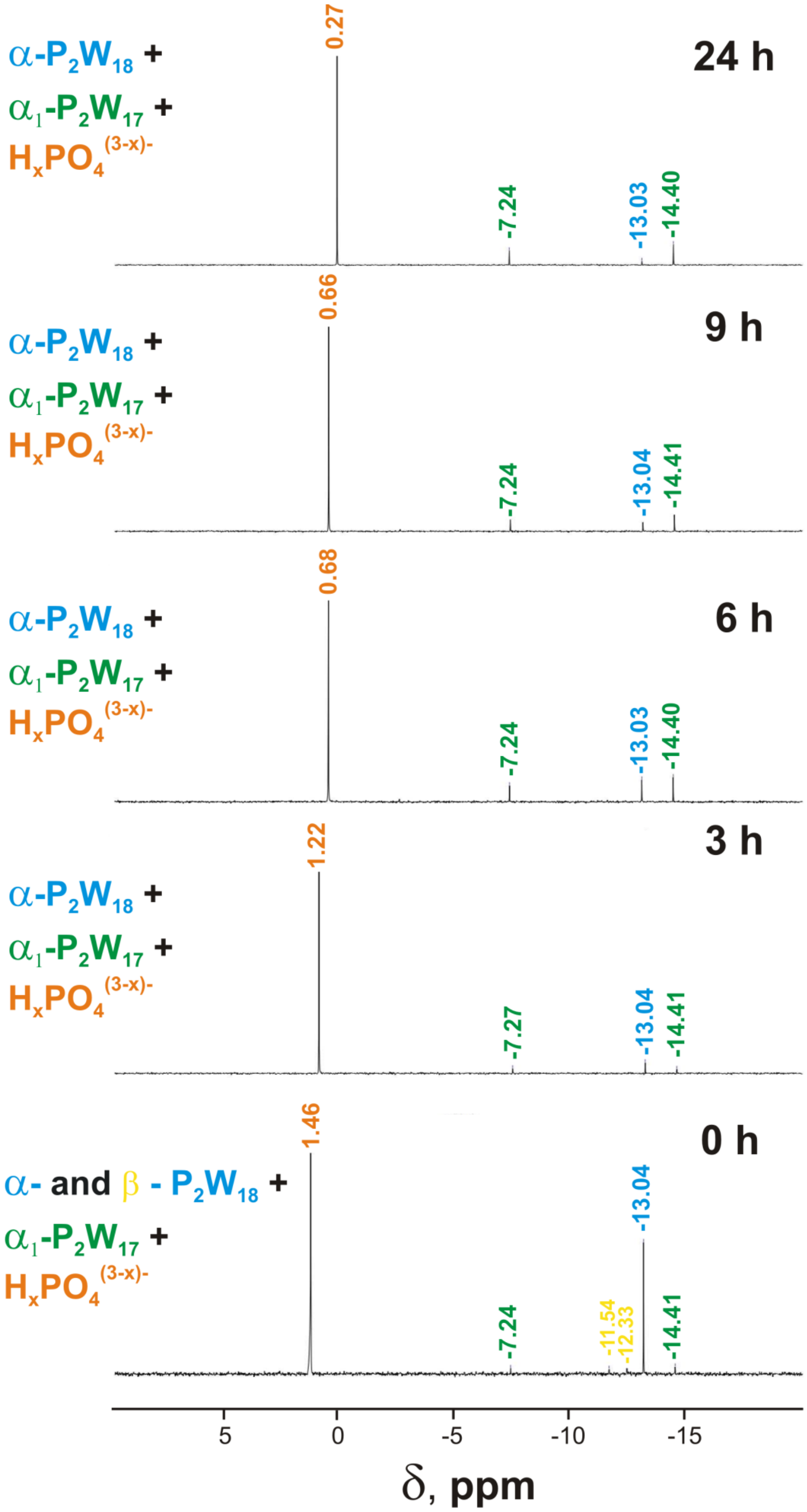

Figure S3. ^{31}P-NMR spectra of **P_2W_{18}** in PBS medium at pH 7.4, recorded after different time intervals of incubation at 37 °C. In phosphate formula x = 0-2.

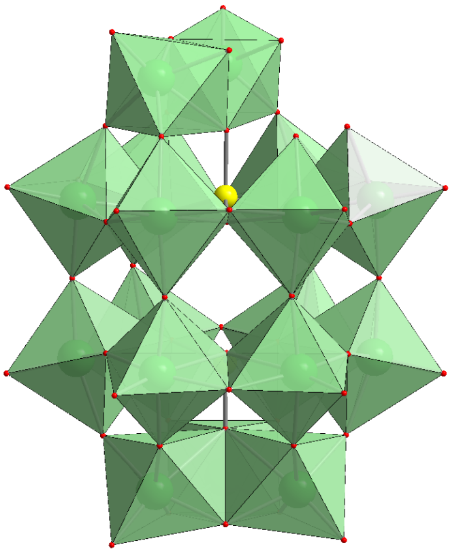

Figure S4. Polyhedral representation of $[P_2W_{17}O_{61}]^{10-}$. Color code: WO_6, mint; P, yellow; O, red.

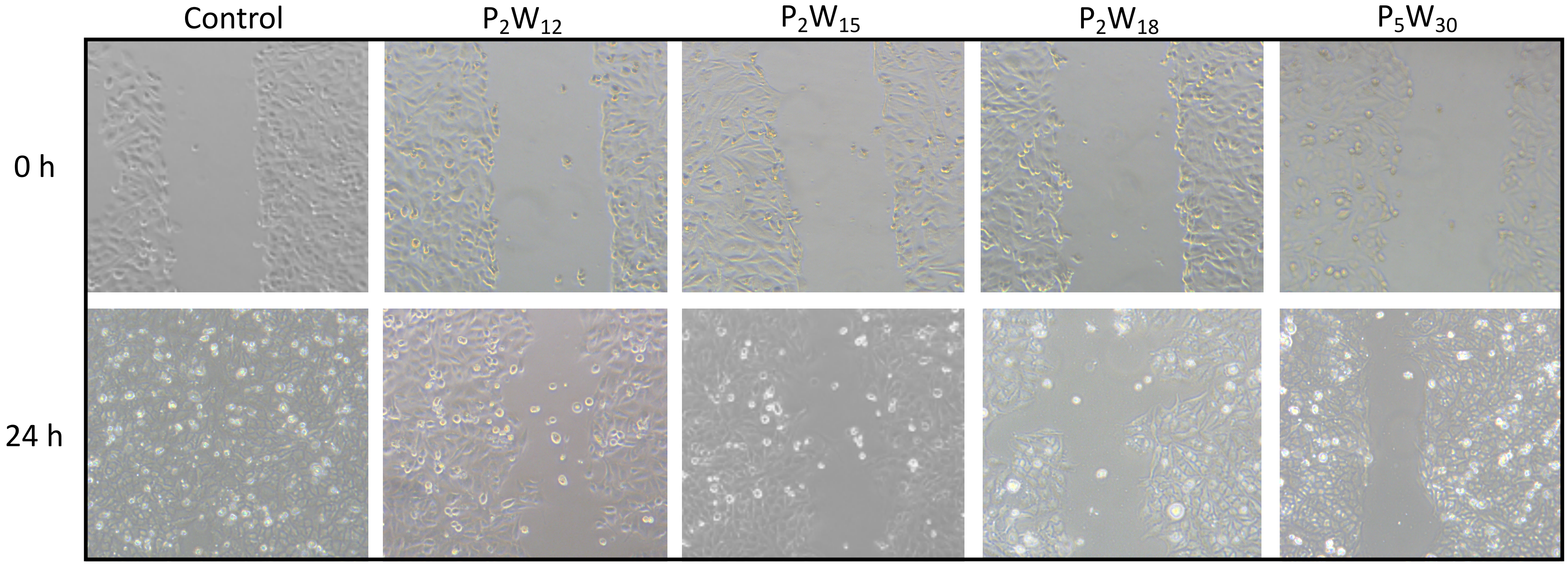

Figure S5. Effect of POTs on cell migration of human melanoma cells. Representative images of wound closure progression in cells non-treated (Control) and treated with 5 µM POTs at 0 and 24 h.
